# Verteporfin Mitigates Isoproterenol-Induced Myocardial Hypertrophy by Attenuating IL-6/STAT3 in Cardiac Fibroblasts

**DOI:** 10.1155/cdr/2852780

**Published:** 2025-08-28

**Authors:** Sunpeng Sheng, Lu Ding, Jinbiao Lai, Zelei Ye, Ru Zhao, Shijia Chen, Jianshe Ma, Junming Fan, Peifeng Jin

**Affiliations:** ^1^Department of Cardiac Surgery, The First Affiliated Hospital of Wenzhou Medical University, Wenzhou, Zhejiang, China; ^2^Institute of Hypoxia Medicine, School of Basic Medical Sciences, Wenzhou Medical University, Wenzhou, Zhejiang, China; ^3^School Infirmary, Shaanxi Institute of Technology, Xian, Shaanxi, China; ^4^Advanced Medical and Dental Institute, Universiti Sains Malaysia, Gelugor, Pulau Pinang, Malaysia

**Keywords:** cardiac fibroblasts, interleukin-6, isoproterenol, myocardial hypertrophy, yes-associated protein

## Abstract

**Background:** Yes-associated protein (YAP) is a major downstream nuclear coactivator of the Hippo pathway and is activated during myocardial hypertrophy. Verteporfin, a YAP inhibitor, may serve as a potential treatment for myocardial hypertrophy.

**Aim:** This study was aimed at exploring the role and underlying mechanisms of verteporfin in isoproterenol (ISO)-induced myocardial hypertrophy both in vivo and in vitro.

**Methods:** GSE18801 directs our focus toward the Hippo pathway role in myocardial hypertrophy. Using an ISO-induced myocardial hypertrophy rat model, YAP expression and localization were observed through Western blot and immunofluorescence. Histopathological analysis was performed to evaluate cardiomyocyte cross-sectional area, and echocardiographic examinations were conducted to assess cardiac function. In vitro, primary neonatal rat cardiomyocytes (NRCMs) were cultured with conditioned medium from cardiac fibroblasts (CF-CM) treated with ISO to observe cell hypertrophy. Mechanistically, GSE203358 dataset analysis, enzyme-linked immunosorbent assay (ELISA), and Western blot were utilized to investigate the effects of ISO and verteporfin on IL-6, STAT3, and p-STAT3 levels in CFs. Subsequently, the changes in the IL-6/STAT3 pathway were evaluated in CFs treated with ISO and verteporfin. Additionally, recombinant IL-6 and IL-6 inhibitor were applied to CMs treated with CF-CM to observe changes in cardiomyocyte size.

**Results:** Verteporfin improved cardiac performance in rats receiving ISO. In cultured NRCM, both ISO and CF-CM treated with ISO could induce cardiomyocyte hypertrophy. Verteporfin did not attenuate ISO-induced cardiomyocyte hypertrophy. However, it could attenuate hypertrophy induced by the CF-CM treated with ISO. GSE203358 indicated the involvement of the IL-6/STAT3 pathway in the presence of verteporfin in CFs. Additionally, verteporfin reduces IL-6 production in cultured CFs subjected to ISO treatment. Notably, the effects of verteporfin on NRCM were reversed by IL-6.

**Conclusions:** Verteporfin protects the heart against ISO-induced myocardial hypertrophy by regulating IL-6/STAT3 in cardiac fibroblasts.

## 1. Introduction

Cardiac hypertrophy, often triggered by inflammation due to environmental stress or external infection, is a key pathological process contributing to heart failure, a severe cardiovascular condition with high morbidity and mortality worldwide [1, 2]. While short-term hypertrophy can serve as a compensatory mechanism to protect cardiac function, persistent hypertrophy leads to an increase in cardiomyocyte (CM) size and disrupts cardiac performance, ultimately resulting in heart failure without timely intervention [3–6]. Understanding the mechanisms driving pathological cardiac hypertrophy is therefore crucial for developing effective therapeutic strategies.

It is well known that the Hippo pathway plays an important biological regulatory function in controlling the organ size in mammals through regulating cellular differentiation, proliferation, and apoptosis, and yes-associated protein (YAP) is a major downstream nuclear transcriptional coactivator of the Hippo pathway [7, 8]. Mechanistically, a growing number of studies have shown that YAP plays critical roles in controlling inflammation and cellular functions through its dephosphorylation and translocation into the nucleus and binds to the TEA domain transcription factors to activate the target gene expression, thereby promoting cellular functions such as proliferation and migration [9–13]. Several recent studies indicated the involvement of Hippo-YAP signaling in controlling the cardiovascular system including cardiac development and angiogenesis [14–16]. MicroRNA-206 mediates YAP-induced cardiac hypertrophy by downregulating FoxP1 [17]. Loss of YAP function causes insufficient cardiac hypertrophy in response to stress, leading to exacerbation of cardiac dysfunction [18]. However, despite significant progress in elucidating the role of YAP in cardiovascular regulation, its roles in myocardial hypertrophy and the potential mechanism need to be further illustrated.

In this study, we utilized the YAP inhibitor verteporfin to regulate YAP activity, aiming to validate the effects of YAP inhibition on myocardial hypertrophy and to explore the underlying mechanisms. Our findings demonstrated that verteporfin protects the heart from ISO-induced myocardial hypertrophy through mechanisms involving the regulation of the IL-6/STAT3 pathway in cardiac fibroblasts (CFs).

## 2. Materials and Methods

### 2.1. Bioinformatics Analysis

#### 2.1.1. Data Sources and Analysis Process

The datasets GSE18801 and GSE203358 included in this study were acquired from the GEO database. This study primarily focuses on the heart samples from three normal group (CON) and three ISO-treated group (ISO) mice in the GSE18801 dataset, as well as the cardiac stromal cell samples from the control group (CTRL) and verteporfin-treated group (VP) in the GSE203358 dataset.

Data processing and visualization were performed using the R language. Initially, the “limma” package was used to identify differentially expressed genes (DEGs) and create volcano plots, with the following filtering criteria: |log2 fold change| > 1.5 and *p* value < 0.05. Subsequently, DEGs were visualized using heatmaps generated by the “heatmap” package. To explore the potential biological roles of DEGs, pathway enrichment analysis was conducted using the Kyoto Encyclopedia of Genes and Genomes (KEGG) database. Additionally, Gene Set Enrichment Analysis (GSEA) was employed to gain a more intuitive understanding of the gene expression levels in highly enriched functional pathways. These processes were implemented through the “clusterProfiler” package, with *p* < 0.05 considered statistically significant.

#### 2.1.2. Construction of the Protein–Protein Interaction (PPI) Network and Identification of Key Genes

A PPI network was constructed using the STRING database for DEGs related to the Hippo signaling pathway, angiogenesis, extracellular matrix (ECM), and inflammation, considering only experimentally validated interactions with a combined score higher than 0.7. The constructed PPI network was then imported into Cytoscape software, and Degree and Betweenness centrality algorithms were applied to evaluate the key genes within the network, adjusting the network diagram to highlight key gene nodes.

### 2.2. Reagents and Antibodies

ISO was purchased from Sigma-Aldrich (St. Louis, Missouri, United States). Verteporfin (A12658) was purchased from Adooq BioScience (Irvine, California, United States). Recombinant Human IL-6 (P5138) was purchased from Beyotime (Shanghai, China). LMT-28 (HY-102084) was purchased from MedChemExpress (New Jersey, United States). ISO was dissolved in normal saline (0.9% NaCl), whereas verteporfin was reconstituted in 5% dimethyl sulfoxide (DMSO). All of the other reagents used in this study were of analytical grade. Antibodies against YAP (#8418), IL-6 (#12153), p-STAT3 (#9145), STAT3 (#12640), GAPDH (#5174), and secondary antibodies were obtained from Cell Signaling Technology (Danvers, Massachusetts, United States).

### 2.3. Animal Experiments

All animals used in this study were purchased from Shanghai SLAC Laboratory Animal Co. Ltd. (Shanghai, China). Briefly, 6-week-aged male Sprague-Dawley rats weighing ~200 g were randomly assigned to four groups as follows: Group I (control): Rats were administered saline intravenously every 3 days and subcutaneous injection of saline once daily for a total of 10 days. Given the influence of estrogen and physiological cycles on cardiovascular diseases, female rats were not included in this experiment. Group II (VP): Rats were administered verteporfin (10 mg/kg, dissolved in saline, Sigma-Aldrich) intravenously every 3 days and subcutaneous injection of saline once daily for a total of 10 days. Group III (ISO): Rats were administered saline intravenously every 3 days and subcutaneous injection of ISO (5 mg/kg/day, dissolved in saline, Sigma-Aldrich) once daily for a total of 10 days. Group IV (ISO + VP): Rats were administered verteporfin intravenously every 3 days and subcutaneous injection of ISO once daily for a total of 10 days.

All experimental protocols of this study were approved by the Institutional Animal Use and Care and the Animal Ethics Committee of Wenzhou Medical University (WYYY-AEC-YS-202300485, China) and complied with the National Institutes of Health Guide for the Care and Use of Laboratory Animals (NIH Publications No. 8023, revised 1996).

### 2.4. Western Blot Assay

Protein extracts (50 *μ*g) from rat hearts or CMs were prepared in a homogenization buffer and then subjected to 10% sodium dodecyl sulfate-polyacrylamide gel electrophoresis. The proteins were then transferred onto a polyvinylidene difluoride membrane (Millipore, Billerica, Massachusetts, United States). The membrane was blocked for 1 h at room temperature with 10% bovine serum albumin in tris-buffered saline. The blots were incubated overnight at 4°C with anti-YAP, anti p-YAP, anti-IL-6, anti-STAT3, and anti-p-STAT3 antibodies and secondary antibodies. Protein expression was visualized using enhanced chemiluminescence reagents (Bio-Rad). Protein levels were analyzed using ImageJ analysis software version 1.38e.

### 2.5. Cardiac Histological Analysis

At the end of the experiment, the animals were euthanized by sodium thiopental injection (100 mg/kg, dissolved in saline, Sigma-Aldrich), and blood samples were collected and centrifuged to separate the serum. The serum was stored at −20°C for subsequent biochemical assays. The hearts were immediately excised and perfused with ice-cold phosphate-buffered saline (PBS). The final body weight (BW) and heart weight (HW) were recorded, and the heart-to-body weight (HW/BW) ratio was calculated and used to estimate the degree of myocardial hypertrophy. A separate cohort of rats was transcardially perfused with PBS, followed by fixation with 4% paraformaldehyde, then the hearts were excised and embedded in paraffin, and the heart sections (5 *μ*m) were prepared and stained with hematoxylin and eosin (H&E). YAP protein in cardiac tissue was detected by immunofluorescence. Briefly, the sections were incubated overnight at 4°C with a primary antibody against YAP, followed by incubation with a secondary antibody. To measure histological changes, cardiac images were observed under a light microscope (Nikon, Tokyo, Japan).

### 2.6. Wheat Germ Agglutinin (WGA) Assay

Cardiac paraffin sections were deparaffinized to water, followed by treatment with 0.1% Triton X-100 as a permeabilizing agent. The samples were then treated with a blocking solution and incubated with Alexa Fluor 488-labeled WGA for 30 min. Finally, the sections were observed under a fluorescence microscope. The cross-sectional area of CMs was quantified using ImageJ software.

### 2.7. Echocardiography Analysis

Cardiac geometry and functional parameters were recorded in anesthetized rats using echocardiographic examinations with the Vevo 2100 system (Fujifilm Visual Sonics, Toronto, Canada), according to a previous study [19]. Left ventricular (LV) end-diastolic posterior wall thickness (LVPWs), interventricular septal thickness (IVSd), and end-diastolic diameter (LVEDD) were recorded from the M-mode images. Echocardiographic parameters, including the LV end-diastolic volume (LVEDV), ejection fraction (LVEF), and fractional shortening (LVFS) were calculated.

### 2.8. Hemodynamic Studies

Following the final drug treatment, LV hemodynamics were measured 24 h later, as previously described [20]. In brief, rats were anesthetized in an isoflurane chamber and then intubated and ventilated with 2% isoflurane. Subsequently, a small incision was made to locate the right carotid artery, through which a microcatheter was inserted and advanced into the LV. Hemodynamic measurements were analyzed using Lab Chart 7 software (version 7.2, ADInstruments, Sydney, Australia). The pressure in the aorta and LV was calculated for all animals under anesthesia. The peak LV systolic pressure (LVSP) and LV end-diastolic pressure (LVEDP) were measured, and the maximal slopes of systolic pressure increment (dP/dt_max_), diastolic pressure decrement (dP/dt_min_), and indexes of contractility and relaxation, respectively, were analyzed (NIH, Bethesda, Maryland, United States).

### 2.9. Enzyme-Linked Immunosorbent Assay (ELISA)

The concentrations of IL-6 (#E-EL-R0044c), atrial natriuretic peptide (ANP) (#E-EL-R0017c), and brain natriuretic peptide (BNP) (#E-EL-R0126c) in the serum, heart, and culture supernatants were measured by commercially available ELISA kits (Elabscience, Wuhan, China). ELISA was performed according to the manufacturer's instructions. All samples were assayed in duplicate.

### 2.10. Cell Culture

Neonatal rat CMs/CFs were isolated and cultured from 1–2-day-old Sprague-Dawley rats, as previously described [21]. Briefly, hearts were harvested from rats euthanized by decapitation following excessive sodium thiopental anesthesia, immersed in PBS, and then minced with scissors. Small pieces of heart tissue were digested with 0.01% collagenase II (Sigma-Aldrich) in PBS at 37°C, and the sample from the first 10 min of digestion was discarded. The isolated cells were mixed with horse serum and centrifuged at 1000 rpm for 5 min. The resulting cell pellet was resuspended in warm Dulbecco's modified Eagle medium (DMEM) containing 5% fetal bovine serum (FBS) and 1% penicillin–streptomycin (P/S) and then preplated and incubated for 30 min at 37°C to allow CFs to adhere to the plate.

The unadhered cells were pelleted again and resuspended in DMEM containing 5% FBS, 1% P/S, and bromodeoxyuridine (1:100, to inhibit fibroblast growth). Subsequently, CMs were plated at a concentration of 1 × 10^6^ cells per 35-mm plate.

### 2.11. Cell Treatments

CFs were categorized into control, VP, ISO, and ISO + VP groups. The control group cells were treated with physiological saline, the VP group underwent incubation with verteporfin (250 nM), the ISO group was exposed to 10 *μ*M ISO, and the ISO + VP group was subjected to a combined treatment of 10 *μ*M ISO and verteporfin (250 nM) for a duration of 24 h.

In an in vitro experiment where CFs treated with ISO were subjected to verteporfin treatment, the levels of IL-6 were assessed through ELISA at various time points (0, 5, 15, 30, 60, and 120 min and 24 h).

### 2.12. Conditioned Medium of CFs (CF-CM) Induces Hypertrophy in Neonatal Rat CMs (NRCMs)

ISO (10 *μ*M) was added into the culture medium for CFs. ISO-treated CFs were incubated in the presence or absence of verteporfin (250 nM) for 12 h. Then, the CF-CM was collected to induce hypertrophy in CMs. The cell cross-sectional area was assessed via Phalloidin staining, while the expression of hypertrophy-related genes was evaluated using RT-qPCR, and IL-6 and STAT3 protein levels were determined by Western blot assay.

In the IL-6 intervention experiment, cells were divided into four groups: DMSO, LMT-28, VP, and VP + IL-6 groups. All interventions were conducted within the CF culture medium. The DMSO group had DMSO incorporated into the medium. The LMT-28 group was supplemented with 30 *μ*M LMT-28 (MCE, HY-102084), an IL-6 inhibitor. The VP group was treated with verteporfin (250 nM), while the VP + IL-6 group received a combination of verteporfin (250 nM) and 50 ng/mL recombinant IL-6 (Abclonal, RP00201) for cultivation. Following 12 h of incubation, the conditioned medium supernatant from these cultures was utilized to further incubate CMs for an additional 12 h. The cross-sectional area of CMs was visualized using Phalloidin staining, and the expression of hypertrophy-associated genes was quantified using RT-qPCR.

### 2.13. Real-Time PCR

Total RNA from primary CMs was extracted using Trizol reagent (Invitrogen, Carlsbad, California, United States) according to the manufacturer's protocol. Reverse transcription was performed using an iScript cDNA synthesis kit (Bio-Rad, Hercules, California, United States), and real-time PCR analysis was performed using SYBR Green (Bio-Rad). The primer sequences including ANP, BNP, and *β*-myosin heavy chain beta (*β*-MHC) were as follows: ANP: F-TGGGGAAGTCAACCCGTCTCAG, R-GCGAGCAGAGCCCTCAGTTTG; BNP: F-GATCTCCAGAAGGTGCTGCC, R-GCAGCTTCTGCATCGTGGA; *β*-MHC: F-AAGTGAAGAGCCTCCAGAGTCTGC, R-GGGCTTCACGGGCACCCTTAGAGC; GAPDH: F-GGCACAGTCAAGGCTGAGAATG, R-ATGGTGGTGAAGACGCCAGTA. Relative gene levels were normalized to the GAPDH level.

### 2.14. Phalloidin Staining

The cell coverslips were fixed with 4% PFA for 30 min. The cells were treated with 0.1% Triton X-100 for 10 min. An appropriate amount of Phalloidin solution was added to the cell samples and incubated in the dark for 60 min. DAPI was used to stain the nuclei. The cell samples were mounted with an antifade reagent. Observations were made using a fluorescence microscope; the cell surface area was estimated based on the distribution of actin filaments.

### 2.15. Statistical Analysis

All data are shown as the mean ± standard error of the mean (SEM). Comparisons between the two groups were analyzed using a two-tailed unpaired *t*-test. Furthermore, comparisons among three or more groups were analyzed using a one-way analysis of variance (ANOVA) followed by Bonferroni's test for multiple comparisons. A *p* value < 0.05 was used to indicate statistical significance.

## 3. Results

### 3.1. ISO Treatment Upregulates YAP Expression in Hypertrophic Rat Hearts

**Figure 1:**
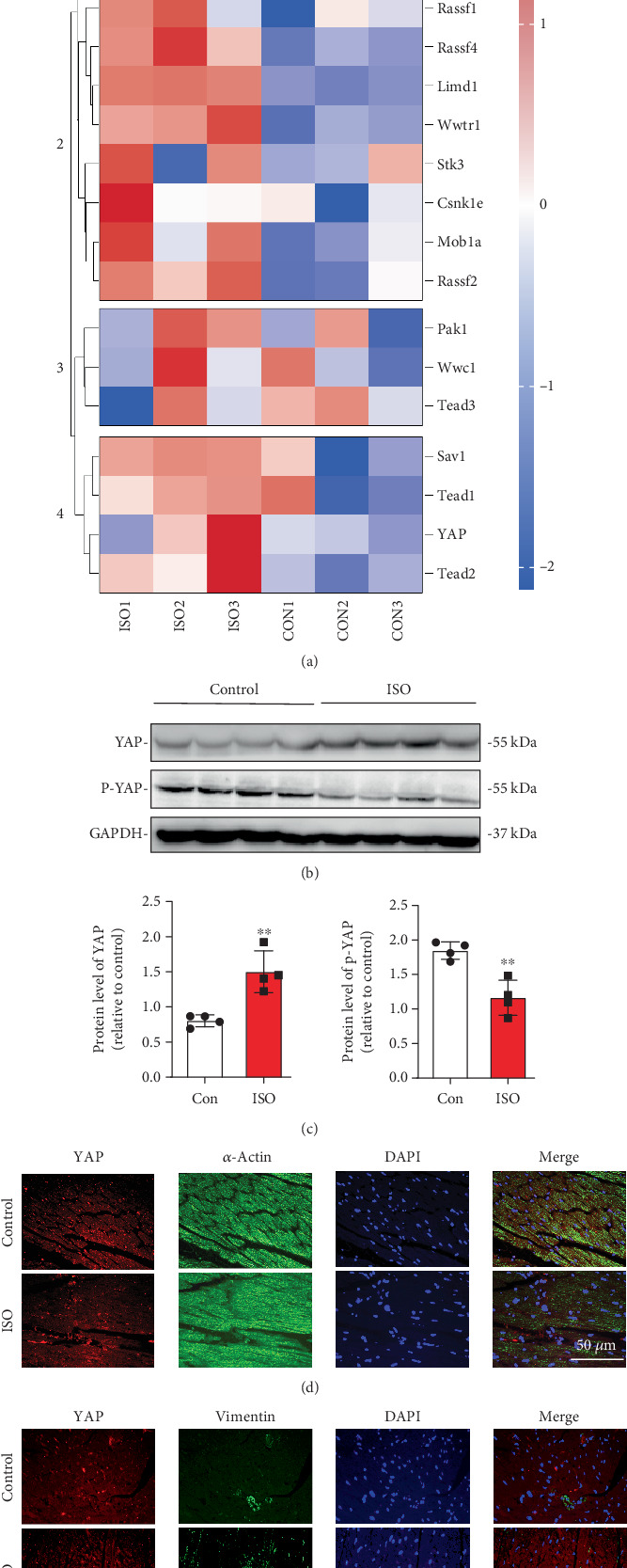
Changes in YAP expression in the hearts of rats following ISO treatment. (a) Differential expression of the Hippo signaling pathway obtained through bioinformatics analysis (GSE18801). (b) Expression of YAP and p-YAP in the heart following ISO treatment, assessed by Western blot. (c) Quantification of Western blot data. Immunofluorescence analysis of YAP expression in cardiac tissue, with (d) CMs marked by *α*-actin and (e) CFs marked by vimentin. ⁣^∗∗^*p* < 0.01, compared with the control group.

DEGs between the control group and the ISO-treated group of rats were subjected to GSEA, revealing that the Hippo signaling pathway was highly expressed in the hearts of ISO-induced myocardial hypertrophy rats, including YAP1 (Figure 1a). To further elucidate the role of YAP1 in myocardial hypertrophy, the changes of YAP in myocardial hypertrophy rats were examined. Western blot assay confirmed that YAP protein levels were significantly increased in ISO-treated rats, while the YAP phosphorylation was significantly decreased (Figure 1b,c). Immunofluorescence colocalization revealed that YAP expression in the myocardial tissue of ISO-induced mice was elevated compared to the control group, with YAP localized in both CMs (stained with *α*-actin) and CFs (stained with vimentin) (Figure 1d,e). Immunofluorescence results showed that the immunoactivity of YAP in cardiac tissue was increased in ISO-treated rats compared with that in control rats (Figure 1d,e).

### 3.2. Verteporfin Treatment Attenuates ISO-Induced Myocardial Hypertrophy and Downregulates ANP and BNP Protein Expression Levels

**Figure 2:**
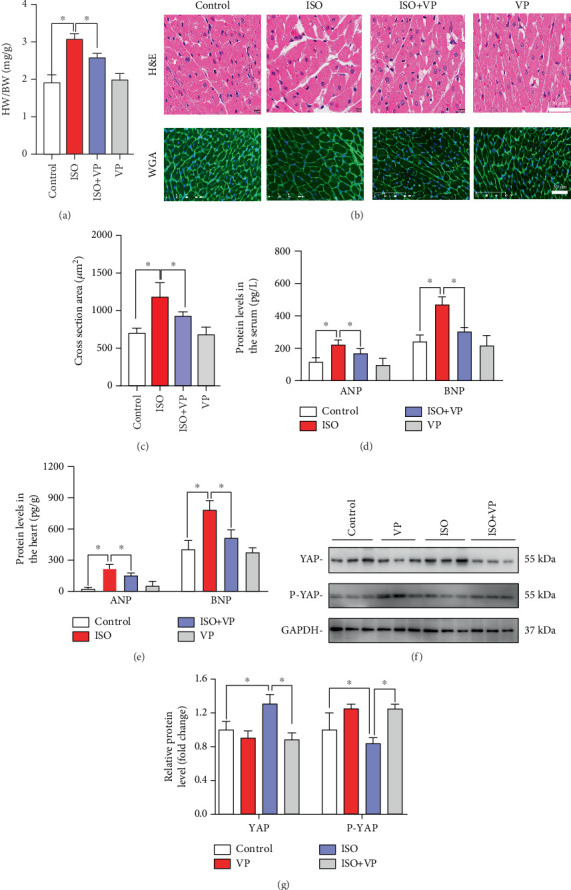
Effects of verteporfin treatment on ISO-induced myocardial hypertrophy. (a) Heart-to-body weight ratio in rats. (b) HE staining (upper); wheat germ agglutinin staining (lower). (c) Cross-sectional area of cardiomyocytes. (d) Levels of ANP and BNP in serum. (e) Levels of ANP and BNP in cardiac tissue. (f) Western blot analysis of YAP and p-YAP protein expression in cardiac tissue. (g) Quantification of YAP and p-YAP protein expression from Western blot results. ⁣^∗^*p* < 0.05, compared with the ISO group.

To elucidate the potential roles of YAP during myocardial hypertrophy induced by ISO as described above, verteporfin, an inhibitor for YAP, was administered and the cardiac structure for myocardial hypertrophy was examined in rat hearts. The HW/BW ratio was significantly decreased in ISO + verteporfin-treated rats compared with that in ISO-treated rats (Figure 2a). Consistently, the cross-sectional area of cardiac tissue was decreased in ISO + verteporfin-treated rats compared with that in ISO-treated rats (Figure 2b,c). Furthermore, the ELISA results showed that the hypertrophic phenotypes in ISO-treated rats were accompanied by the upregulation of hypertrophic genes, including ANP and BNP, both in the peripheral serum (Figure 2d) and hearts (Figure 2e). However, ISO + verteporfin downregulated ANP and BNP levels in serum and hearts compared with those in ISO-treated rats.

Western blot analysis demonstrated that treatment with verteporfin alone did not significantly alter the levels of YAP or p-YAP in myocardial tissue. In contrast, ISO treatment markedly increased YAP expression and significantly reduced p-YAP levels in myocardial tissue. In the ISO + VP group, YAP levels were significantly lower than in the ISO group, while p-YAP levels were significantly higher than in the ISO group (Figure 2f,g).

### 3.3. Verteporfin Treatment Ameliorates ISO-Induced Heart Dysfunction

**Figure 3:**
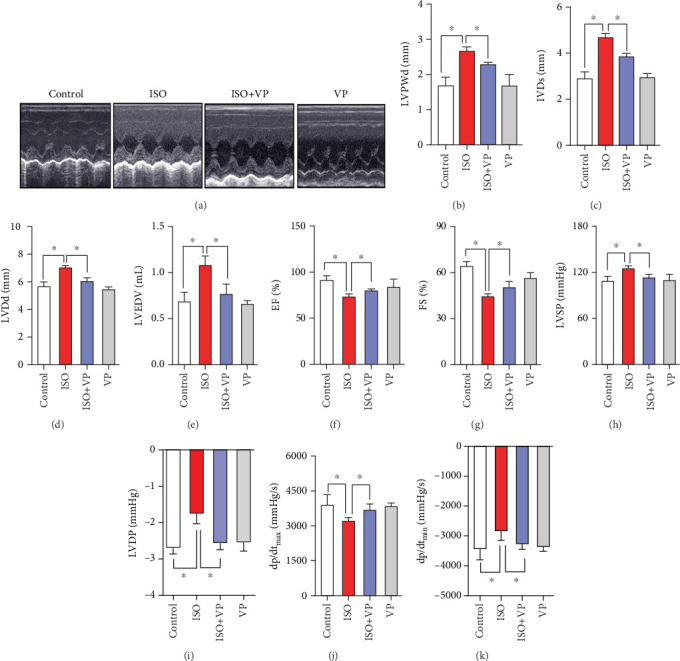
Verteporfin alleviates cardiac dysfunction in ISO-induced myocardial hypertrophy rats. (a) Echocardiographic examinations; (b) left ventricular posterior wall end-diastolic thickness (LVPWd); (c) left ventricular end-systolic diameter (LVDs); (d) left ventricular end-diastolic diameter (LVDd); (e) left ventricular end-diastolic volume (LVEDV); (f) ejection fraction (EF); (g) fraction shortening (FS); (h) the peak left ventricular systolic pressure (LVSP); (i) left ventricular end-diastolic pressure (LVDP); (j) diastolic pressure decrement (dp/dt_min_); (k) maximal slopes of systolic pressure increment (dp/dt_max_). ⁣^∗^*p* < 0.05, compared with the ISO group.

To further explore the role of YAP in ISO-induced myocardial hypertrophy, the rats were injected with verteporfin (i.p.) and subjected to functional parameters. ISO caused echocardiography-detectable dysfunction, as evidenced by an abnormal electrocardiogram curve (Figure 3a), an increase in cardiac function parameters including left ventricular posterior wall end-diastolic thickness (LVPWd), left ventricular end-systolic diameter (LVDs), left ventricular end-diastolic diameter (LVDd), LVEDV, the peak LVSP, left ventricular end-diastolic pressure (LVDP), and diastolic pressure decrement (dp/dt_min_). Besides, ISO caused a decrease in ejection fraction (EF), fraction shortening (FS), and maximal slopes of systolic pressure increment (dp/dt_max_) (Figures 3b, 3c, 3d, 3e, 3f, 3g, 3h, 3i, 3j, and 3k). Remarkably, all these changes induced by ISO treatment were reversed in the presence of verteporfin.

### 3.4. Verteporfin Significantly Improves CM Hypertrophy Induced by CF-CM

**Figure 4:**
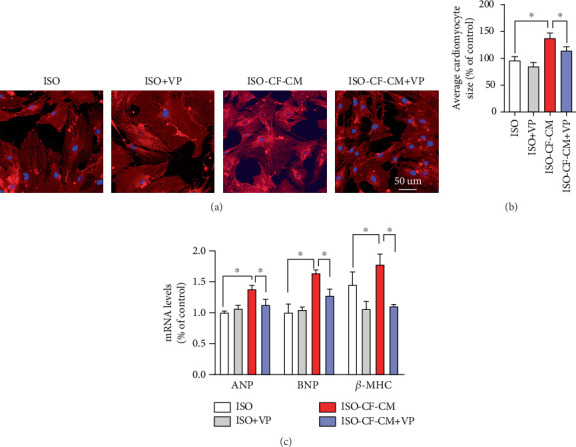
Effects of verteporfin on fibroblast-conditioned medium-induced cardiomyocyte hypertrophy. (a) Phalloidin staining (red indicates actin filaments, blue indicates cell nuclei). (b) Average cross-sectional area of cardiomyocytes. (c) PCR analysis of ANP, BNP, and *β*-MHC mRNA expression in cardiomyocytes. ⁣^∗^*p* < 0.05, compared with the ISO-CF-CM group.

CMs were directly stimulated with ISO and treated with verteporfin to investigate the effect of verteporfin on CM hypertrophy. In parallel, considering the regulatory crosstalk between CFs and CMs, CMs were incubated with conditioned medium derived from ISO-treated CFs to determine whether verteporfin exerts its modulatory effects indirectly via CFs or directly on CMs. Supporting Information 2: Figure S1 illustrates myocardial hypertrophy following ISO treatment. After ISO direct stimulation of CMs, verteporfin treatment did not show a significant improvement in CM hypertrophy. However, compared to ISO-induced hypertrophy, verteporfin treatment exhibited a more pronounced ameliorative effect on hypertrophy induced by CF-CM, as evidenced by a significant reduction in NRCM surface area (Figure 4a,b) and a significant decrease in the expression of ANP, BNP, and *β*-MHC (Figure 4c).

### 3.5. Verteporfin Effect on Myocardial Hypertrophy via Targeting IL-6-STAT3 Pathway in CFs

**Figure 5:**
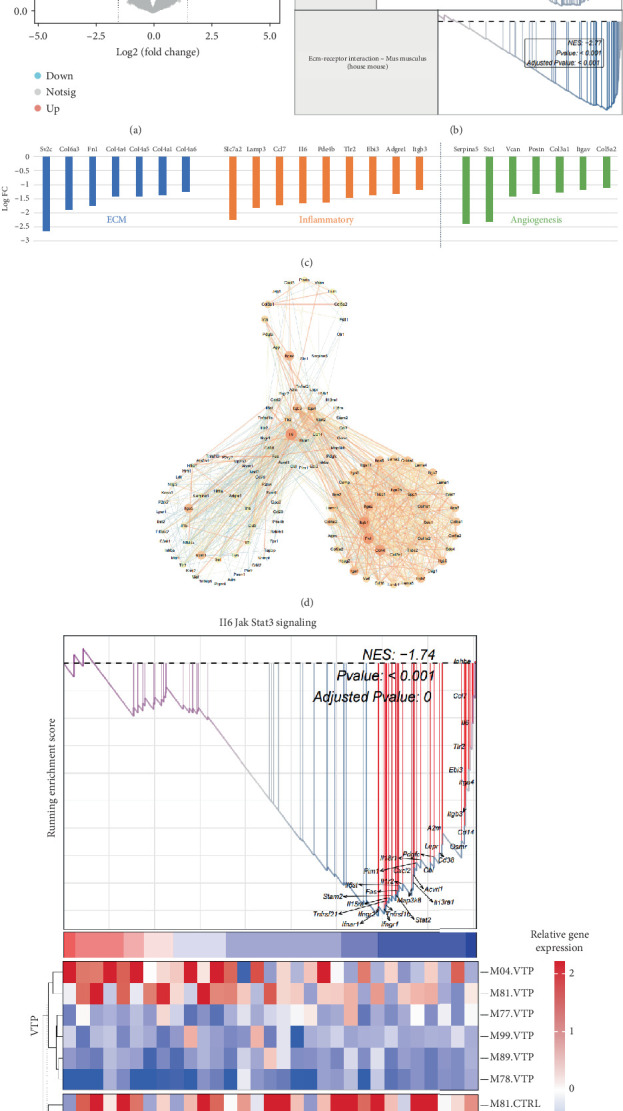
GSEA of pathway enrichment in DEGs of fibroblasts before and after verteporfin treatment. (a) Volcano plot of DEGs in cardiac stromal cells from control and verteporfin-treated mice based on RNA sequencing analysis (GSE203358). (b, c) KEGG pathways enriched by GSEA. Genes related to ECM-receptor interaction, inflammation, and angiogenesis were downregulated upon verteporfin treatment. (d) PPI analysis of genes related to the three biological processes. (e) GSEA of verteporfin's impact on the IL-6-STAT3 pathway.

To identify the targets of verteporfin in myocardial hypertrophy, we conducted RNA-sequencing analysis (GSE203358) to evaluate transcriptomic changes caused by verteporfin in cardiac stromal cells. A total of 540 DEGs were identified, including 321 upregulated and 219 downregulated genes, based on the criteria of |log2 fold change| > 1.5 and *p* value < 0.05 (Figure 5a and Supporting Information 1: Table S1). GSEA of hallmark gene sets and KEGG analysis revealed that upon verteporfin treatment, genes related to inflammation, angiogenesis, and ECM-receptor interaction processes were significantly downregulated (Figure 5b,c). Representative genes involved in these processes include SLC7A2, LAMP3, CCL7, IL-6, PDE4B, TLR2, EBI3, ADGRE1, and ITGB3 (inflammation); Serpin A5, STC1, VCAN, POSTN, COL3A1, ITGAV, and COL5A2 (angiogenesis); and SV2C, COL6A3, FN1, COL4A4, COL4A5, COL4A1, and COL4A6 (ECM remodeling). PPI analysis revealed IL-6 as a central node among genes involved in these biological processes (Figure 5d). Additionally, GSEA demonstrated that verteporfin significantly inhibits the IL-6-STAT3 pathway by downregulating core components such as IL-6 and gp130 (Figure 5e).

### 3.6. Verteporfin Treatment Attenuates ISO-Induced IL-6 Expression in CFs

**Figure 6:**
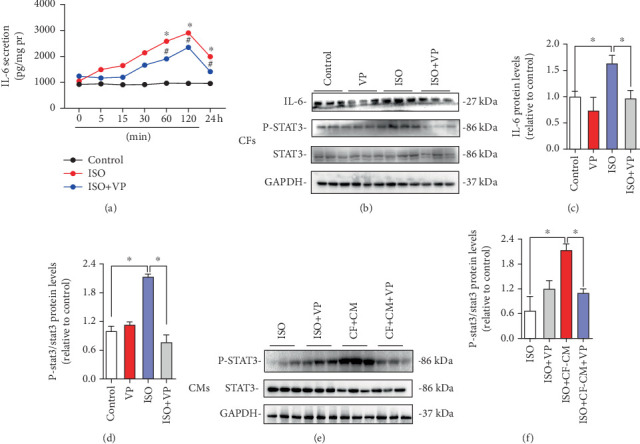
Verteporfin effect on myocardial hypertrophy via targeting the IL-6-STAT3 pathway in cardiac fibroblasts. (a) ELISA analysis of IL-6 protein levels in the culture medium supernatant at 0, 15, 30, 60, and 120 min and 24 h. ⁣^∗^*p* < 0.05, compared with the control group; ^#^*p* < 0.05, compared with the ISO group. (b) Western blot analysis of IL-6, STAT3, and p-STAT3 protein expression in CFs. (c) Quantification of IL-6 from Western blot results. (d) Quantification of p-STAT3/STAT3 from Western blot results. (e) Western blot analysis of STAT3 and p-STAT3 protein expression in CMs. (f) Quantification of p-STAT3/STAT3 from Western blot results. ⁣^∗^*p* < 0.05, compared with the ISO group or the ISO + CF-CM group.

To further explore the regulatory role of verteporfin on IL-6 in CFs, CFs treated with ISO were subjected to verteporfin treatment in vitro. ELISA results further confirmed that IL-6 protein levels were significantly increased in ISO-treated CF culture medium supernatant after 2 h (Figure 6a).

Consistently, Western blotting results showed that IL-6 protein levels and p-STAT3/STAT3 ratio were significantly increased in ISO-treated CFs. However, verteporfin treatment significantly reversed the increase in IL-6 protein expression and p-STAT3/STAT3 ratio induced by ISO (Figures 6b, 6c, and 6d).

Following ISO stimulation of CMs, no significant changes in STAT3 phosphorylation were observed. However, incubation with CF-CM treated with ISO resulted in a significant elevation of STAT3 phosphorylation, which was subsequently suppressed by verteporfin treatment (Figure 6e,f).

### 3.7. Recombinant IL-6 Attenuates the Protective Effect of Verteporfin on CF-CM-Induced CM Hypertrophy

**Figure 7:**
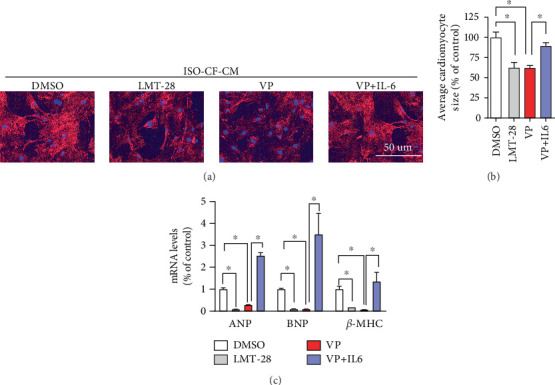
Effects of recombinant IL-6 on CF-CM-induced cardiomyocyte hypertrophy. (a) Phalloidin staining to observe the effect of IL-6 on verteporfin-mediated alleviation of cardiomyocyte hypertrophy. (b) Average cross-sectional area of cardiomyocytes. (c) RT-qPCR analysis of changes in ANP, BNP, and *β*-MHC expression. ⁣^∗^*p* < 0.05, compared with the LMT-28 group or the VP group.

To investigate whether verteporfin mediates the effect of IL-6 in promoting CM hypertrophy, CFs were treated with LMT-28 and recombinant IL-6, respectively, followed by the use of their conditioned medium supernatant to culture CMs. Compared to the DMSO group, suppression of IL-6 by LMT-28 significantly reduced cell surface area, as did treatment with verteporfin. In contrast, the cell surface area in the VP + IL-6 group was significantly elevated compared to the VP group (Figure 7a,b). The mRNA levels of hypertrophy marker genes ANP, BNP, and *β*-MHC were similarly decreased following intervention with LMT-28 or verteporfin. However, in the VP + IL-6 group, the mRNA levels of these markers were significantly higher than those in the VP group (Figure 7c).

## 4. Discussion

This study delves into the role of YAP in ISO-induced cardiac hypertrophy and how it exerts this effect through the regulation of the IL-6/STAT3 signaling pathway in CFs. Additionally, we further investigate the potential protective effects of the YAP inhibitor verteporfin in mitigating ISO-induced cardiac hypertrophy.

Previous studies have emphasized the importance of the YAP signaling pathway in cardiac hypertrophy [22]. Both in tissue samples from hypertrophic cardiomyopathy patients and in the transverse aortic constriction mouse model, YAP activity is significantly elevated compared to normal tissue and mice at both mRNA and protein levels. Notably, CM-specific YAP transgenic mice exhibit phenotypes similar to hypertrophic cardiomyopathy. In vitro experiments with primary CMs from mice show that overexpression of YAP induces cell enlargement [23]. In our analysis of the GSE18801 database, along with the study of YAP expression in the hearts of ISO-treated rats, we confirmed that under ISO-induced conditions, YAP expression is elevated in both CFs and CMs. This finding aligns with previous studies and further solidifies YAP's critical role as a core component of the Hippo signaling pathway in cardiac hypertrophy, suggesting that YAP may be an important target for inducing cardiac hypertrophy.

To evaluate the potential therapeutic effects of verteporfin in alleviating ISO-induced pathological cardiac hypertrophy, we intraperitoneally administered the drug to rats and observed changes in their cardiac function and structure. The results demonstrated that verteporfin significantly improved heart dysfunction and reduced CM cross-sectional area in ISO-treated rats, indicating its potential in alleviating cardiac hypertrophy. Building upon this, we further explored the mechanisms underlying these effects. In the pathogenesis of hypertrophic cardiomyopathy, the paracrine effects of CFs play an important role in stimulating CMs. Research suggests that CFs may negatively impact the structural and electrophysiological differentiation of CMs [24]. Using in vitro cultured CMs, we applied two methods—direct ISO treatment and conditioned medium from ISO-stimulated CFs—to induce CM hypertrophy and compared verteporfin's effects on hypertrophy in both conditions. The results showed that verteporfin had a more pronounced effect on improving hypertrophy induced by conditioned medium from ISO-stimulated CFs in NRCMs. Therefore, we hypothesize that verteporfin exerts its effects by modulating the paracrine activity of CFs on CMs.

Further analysis using microarray data from the GSE203358 database and GSEA revealed that verteporfin treatment improves processes in CFs such as ECM-receptor interaction, inflammation, and angiogenesis. PPI analysis of these processes identified IL-6 as a central player. GSEA further revealed significant alterations in the IL-6-STAT3 signaling pathway in CFs treated with verteporfin. This finding was corroborated in vitro, where IL-6-STAT3 expression was significantly reduced in CFs treated with verteporfin. Studies have shown that IL-6 secreted by CFs promotes CM enlargement through paracrine signaling [25]. Our study also found that exogenous IL-6 partially reversed verteporfin's improvement of CF-CM-induced CM hypertrophy, suggesting that CF-derived IL-6 may mediate the hypertrophic effect induced by YAP.

In conclusion, our study suggests that the YAP inhibitor verteporfin improves ISO-induced pathological cardiac hypertrophy, likely through the inhibition of the IL-6-STAT3 signaling pathway in CFs. This finding provides new insights and potential drug targets for the treatment of cardiac hypertrophy.

## Data Availability

The data that support the findings of this study are available from the corresponding author upon reasonable request.

## Supporting Information

Supporting Information 1 Table S1. List of differentially expressed genes in cardiac stromal cells treated with verteporfin identified by RNA sequencing (GSE203358).

Supporting Information 2 Figure S1. Myocardial hypertrophy following ISO treatment. Phalloidin staining was used to visualize cytoskeletal organization, with red indicating F-actin filaments and blue indicating cell nuclei (DAPI).
