# Supplementary figures and images for: Verteporfin Mitigates Isoproterenol-Induced Myocardial Hypertrophy by Attenuating IL-6/STAT3 in Cardiac Fibroblasts
Source: Cardiovasc Ther. 2025 Aug 28;2025:2852780. doi: 10.1155/cdr/2852780 (PMC12411024; doi:10.1155/cdr/2852780)

## Slide 1
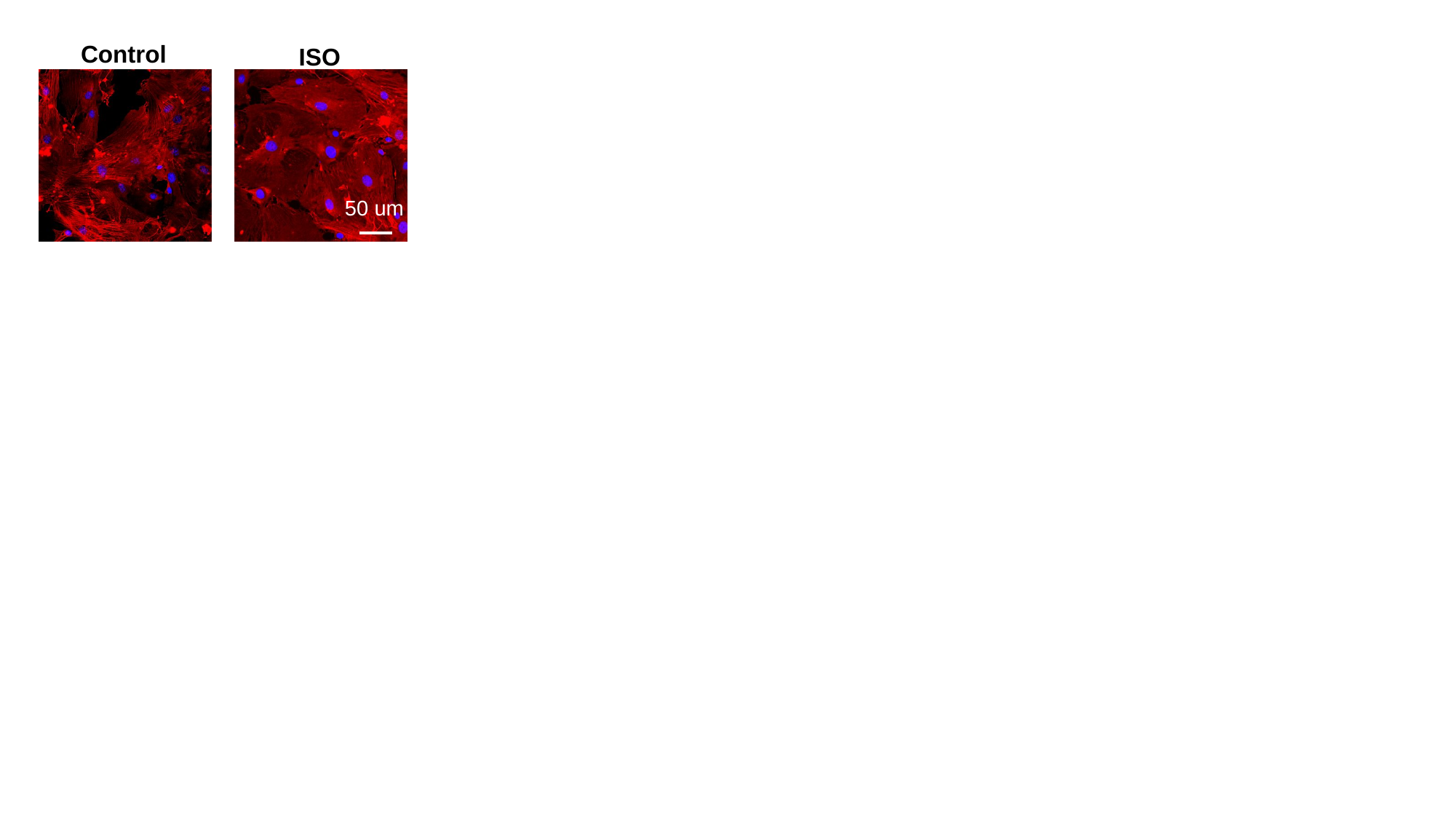

Control
ISO
50 um

Supplement: Supporting Information 2 — Figure S1. Myocardial hypertrophy following ISO treatment. Phalloidin staining was used to visualize cytoskeletal organization, with red indicating F-actin filaments and blue indicating cell nuclei (DAPI). [file 2852780.f2.pptx]
